# Removal of Phenolic Compounds from Water Using Sewage Sludge-Based Activated Carbon Adsorption: A Review

**DOI:** 10.3390/ijerph14101094

**Published:** 2017-09-21

**Authors:** Nuhu Dalhat Mu’azu, Nabeel Jarrah, Mukarram Zubair, Omar Alagha

**Affiliations:** 1 Environmental Engineering Department, University of Dammam, Dammam 31451, Saudi Arabia; najarrah@iau.edu.sa (N.J.); mzzubair@iau.edu.sa (M.Z.); oaga@iau.edu.sa (O.A.); 2 Chemical Engineering Department, Mutah University, Karak 61710, Jordan

**Keywords:** phenolic compounds, wastewater treatment plants, sewage sludge management, activated carbon production, activated carbon adsorption, pollution control

## Abstract

Due to their industrial relevance, phenolic compounds (PC) are amongst the most common organic pollutants found in many industrial wastewater effluents. The potential detrimental health and environmental impacts of PC necessitate their removal from wastewater to meet regulatory discharge standards to ensure meeting sustainable development goals. In recent decades, one of the promising, cost-effective and environmentally benign techniques for removal of PC from water streams has been adsorption onto sewage sludge (SS)-based activated carbon (SBAC). This is attributed to the excellent adsorptive characteristics of SBAC and also because the approach serves as a strategy for sustainable management of huge quantities of different types of SS that are in continual production globally. This paper reviews conversion of SS into activated carbons and their utilization for the removal of PC from water streams. Wide ranges of topics which include SBAC production processes, physicochemical characteristics of SBAC, factors affecting PC adsorption onto SBAC and their uptake mechanisms as well as the regeneration potential of spent SBAC are covered. Although chemical activation techniques produce better SBAC, yet more research work is needed to harness advances in material science to improve the functional groups and textural properties of SBAC as well as the low performance of physical activation methods. Studies focusing on PC adsorptive performance on SBAC using continuous mode (that are more relevant for industrial applications) in both single and multi-pollutant aqueous systems to cover wide range of PC are needed. Also, the potentials of different techniques for regeneration of spent SBAC used for adsorption of PC need to be assessed in relation to overall economic evaluation within realm of environmental sustainability using life cycle assessment.

## 1. Introduction

A number of industrial processes produce huge amount of wastewater contaminated with different organic and inorganic substances. Due to their industrial relevance, PC are amongst the most common organic pollutants found in many industrial wastewaters. The use of PC as antioxidants, flavoring agents and in many other industrial applications offering health benefits and many other opportunities in human endeavors are well documented [1]. However, under certain environmental conditions and/or above certain concentrations, some PC are susceptible to possess significant varying health impacts on humans and other living organisms, even at low concentrations [2]. Due to their high solubility and also volatility, exposure to PC in water and wastewater via nasal, oral, eye or skin and dermal contact can cause harmful effects, with higher levels of exposure even causing eventual death [3]. Additionally, the presence of some of these compounds in water and wastewater can lead to further formation of more harmful byproducts during chemical treatment processes, thereby exacerbating their environmental and health impacts [3]. Moreover, mutagenic and carcinogenic toxicity effects toward humans and other living organisms have been directly attributed to many PC [2].

Consequently, the removal of PC from water and wastewater streams has become an integral component of environmental sustainability. For that, several physicochemical technologies have been proposed for the removal of PC from aqueous streams [3]. In recent decades, one of the promising, cost-effective and environmentally benign options for removal of PC from water streams is adsorption onto SBAC. This is attributed to the excellent adsorptive characteristics of SBAC and also the approach serves as a strategy for sustainable management of huge quantities of SS that are in continual production globally. In fact, SBAC can possess uptake capacity for PC exceeding that of some commercially available GAC [4]. Thus, this option becomes a catalyst to ensuring environmental sustainability, which could allow offsetting increasing cost of sewage sludge waste disposal costs [5]. Recently, as result of abundance and diversity of SS, low cost of SBAC production processes and ease of operational conditions, SBAC adsorption of PC has been receiving tremendous attention from researchers [6,7,8,9,10]. Hence, this paper focuses on reviewing the processes of production of SBAC and their utilization for the removal of PC from water. A wide range of topics are covered, which include SBAC production processes, the physicochemical characteristics of SBAC, factors affecting PC adsorption onto SBAC and their uptake mechanisms and regeneration potentials of the spent SBAC. Finally, recommendations for future studies are also suggested.

## 2. Classifications of Phenolic Compounds

**Figure 1:**
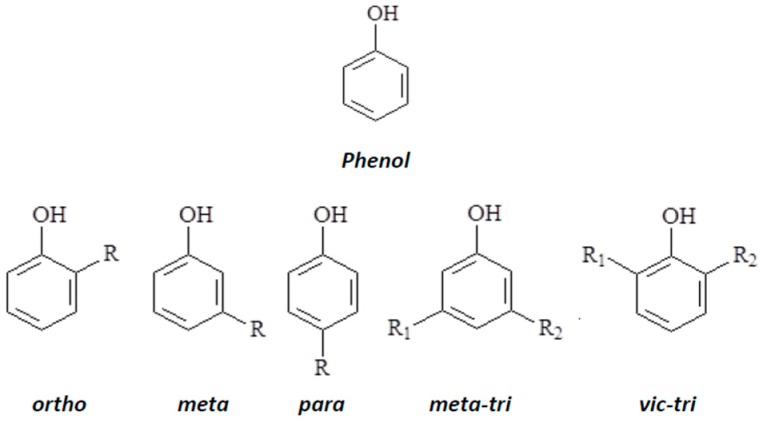
Structure and nomenclature for phenol and substitution patterns of phenolic compounds.

Phenol and its derivatives, collectively known as phenolic compounds are compounds possessing an aromatic ring(s) attached to one or more hydroxyl group (OH). They belong to a variety of groups of chemical substances naturally existing in many living tissues of plants and are also synthesized at lower concentrations by microorganisms [11]. Similarly, a number of them are synthesized industrially for variety of domestic and industrial applications as components of drugs, polymers, dyes and many other organic chemicals [2]. The structure of phenol, the parent compound, has an aromatic benzene ring structure attached to an OH (Figure 1). The entire PC are based on phenol. These compounds can be classified according to Harborne and Simmonds [11] into groups based on the number of carbons in the molecule ranging from the monomers, the simple substituted PC (consisting of the parent phenol attached to one or two more other functional groups) to the more complex β-cyanin lignans, neolignan dimers/oligomers, lignin polymers, tannin oligomers and their polymers, flavanols, quinones, phlobaphenes and polymers [11]. General structures and nomenclature patterns of simple PC (i.e., monomers) are given in Figure 1 with R, R_1_ and R_2_ as generic substituents.

## 3. Production of Sewage Sludge and Its Potential Use as an Adsorbent

Activated sludge systems (ASS) are one of the key treatment processes for wastewater steams employed by a majority of municipal and industrial WWTPs [12]. ASS is a biological process that oxidizes biological and nitrogenous substances as well as removes nutrients in the wastewater and concentrates the residue into a solid mass called sludge. Owing to world rapid population growth as well as industrialization, the quantity of wastewater production has been dramatically increasing; thereby significantly increasing the volume of SS produced globally [13,14]. The SS management process from dewatering till disposal is estimated to represent approximately 25–65% of the overall operating cost of a secondary WWTP [15]. Currently, most of the SS is disposed of via composting for farmland utilization, incineration and land filling [16]. However, the application of sludge for farmland may cause the transfer of pathogens, viruses, heavy metals and organochlorine residues to the crops, with potential transport in the food chain to humans [17]. Due to the strict controls required for incineration of SS as result of its high potential for pollutant release, incineration usually faces stakeholder rejection [9,18]. Competition and growing costs of land space as well as emerging stricter environmental legislations pose a challenge to continual land-filling SS [9,18]. All this suggests the need for devising environmentally sustainable alternatives for the management of SS biomass.

One promising option with high potential of achieving this goal is transforming SS into GAC and its utilization in environmental pollution control [10,19,20,21]. This owes to the fact that effective decontamination of air, water and wastewater containing wide range of harmful substances using GAC has been widely achieved coupled with the process design simplicity and ease of operation [12]. However, commercial GAC are still expensive, especially for large-scale industrial application, thereby jeopardizing the potential of large scale use of the GAC technology [5,12]. Therefore, experts have been expending great efforts to produce GAC from a range of naturally available carbonaceous materials. Given the high carbonaceous nature of sludge, the major requirements for GAC, production of SBAC has been an interesting option in recent years [5,10]. Moreover, the literature has indicated that the gross cost estimate of SBAC possessing high adsorptive capacity is between 5–10% compared to the cost of a standard commercial GAC [10,22]. Thus, this low production cost of SBAC renders them attractive adsorbents for removal of pollutants from water.

## 4. SBAC Production Processes

Sludge derived from WWTP has been receiving increased attention as one of the major identified raw materials for GAC production in recent years [9,10,20,21,22]. This is basically due to three main reasons. The first is the ever increasing population growth coupled with industrialization and urbanization which has led to WWTPS production continually increasing in every community. Second, the huge volume of sludge generation necessitates better alternative sustainable management strategies for meeting stricter effluent discharge and sludge disposal legislations. Lastly, the physicochemical characteristics of raw sludge from different sources (as shown in Table 1) mean SBAC possesses excellent adsorption performance (compared with some commercial GACs). Achievable high surface area and micropore proportion combined with hydrophilic and high-activity oxygen-containing functional groups [23] render SBAC attractive materials for the removal of pollutants from water and wastewater streams. Hence, a literature review indicated different preparation methods as well as several types of sewage sludge biomass were employed for the production of SBAC for PC uptake. Also, different types of municipal and industrial sewage sludge such as DRAWS, DMADS, DUSS, DAEDS etc., were used, thus, the different SBAC produced also exhibited divergent physicochemical characteristics as well as adsorption potentials for the removal of pollutants from water. The process of SBAC production from raw WWTP sewage sludge used for PC uptake generally consists of sludge dewatering and drying which are followed by GAC production which involves two processes: pyrolysis and activation. These processes are discussed in details in the following subsections.

### 4.1. Drying of Dewatered Sludge and Its Pyrolysis and Carbonization

Dewatering and drying are two pre-treatment processes for a raw sludge collected from the Wastewater treatment plant (WWTP). Dewatering is the process of removing water content of a raw sludge, thereby transforming it into a high solid biomass material called “sludge cake”. Dewatering processes that are commonly used include centrifuges, vacuum filters and belt presses. Filter press and centrifuge techniques are the most common and efficient dewatering treatments used in most WWTP, and can produce a total solids concentration of up to 35% as shown in Table 2 [14,21,32]. Drying simply involves heating to evaporation and removing water in order to reduce the moisture of raw sludge below that achievable by conventional dewatering methods [33]. Pyrolysis of dewatered sewage sludge in an inert atmosphere at 400–1000 °C is usually undertaken in order to release volatile components from the dewatered and dried sludge to produce char [25,34]. The main characteristics of the produced char, (i.e., S_BET_ and pore volume) depend primarily on the activation time and temperature during pyrolysis.

Different studies were done to find out the optimum temperature [25,35,36] and activation time [25,37] in order to maximize S_BET_ and the pore volume with reported variation optimum conditions. Carbonization parameters and precursor physiochemical properties significantly influence the textural and other important characteristics of the produced SBAC. Therefore it is important to optimize the operating conditions to achieve the best adsorptive properties of the final carbonized material. Rio et al. [25] conducted the carbonization of two different sludges (normal and the other containing 40% lime) and found that increase in carbonization temperature yielded a higher surface area and micropore volume. They attributed this improvement to a reduction in the number of acidic functional groups and an increase in the number of basic functional groups with increasing temperature. Other studies have reported similar observations [35,38]. In addition, the physiochemical properties of sludge (Table 1), particularly the ash content, have a significant impact on the textural properties of the carbonized activated carbon. Mansalvo [39] carbonized a sludge of S_BET_ (3 m^2^/g) at different temperatures (450, 600 and 750 °C) and attained the maximum S_BET_ (44 m^2^/g) at 750 °C. The resultant carbonized sludge was rich in carbon content which was due to the evolution of volatiles during carbonization [40,41]. However, the low S_BET_ and dominant mesoporous structure was attributed to high ash content of sludge that limits the development of microporous structures. In another study, Julcour Lebigue et al. [42] carried out carbonization of two sludges with ash contents of 20% and 40% at 900 °C among which which the latter yielded the highest S_BET_ of 180 m^2^/g. Numerous studies have also corroborated similar behaviors [43,44].

### 4.2. Physically Activated SBAC

The methods to activate chars produced by pyrolysis can be simply classified into physical activation and chemical activation methods. Physical activation involves burning-off the carbon fraction of the dried sewage sludge. However, the inorganic content of the sludge is usually high and consequently, activated carbon that has been produced by physical activation usually exhibits relatively low surface area [21]. Table 3 shows the textural properties (S_BET_, micro pore, mesoporous volume and pH) of physically activated SBACs and their uptake capacity of phenolic compounds. These characteristics depend mainly on the activating gas type of used, temperature and activation time of the activation process. Different physical activating gases, air [40], CO_2_ [45,46] and steam [47,48,49,50] were investigated by different researchers.

Data obtained regarding S_BET_ suggests that the best physical activation was steam activation with the highest achieved S_BET_ of 226 m^2^/g [53,54]. Using response surface methodology, S_BET_ was maximized at an activation temperature of 763 °C and an activation period of 39 min. However, the conditions were 790 °C and 70 min to maximize the pore volume [49,50]. The textural properties of SBAC by using physical activation (steam, CO_2_ and air) are given in Table 3.

Several studies have reported that activation using steam, CO_2_ and air can significantly improve the textural properties of SBAC. Marques et al. [53] used steam and carbonization activation from which they concluded that steam activation is effective in producing high S_BET_ (179.3 m^2^/g). Moreover, the pre-carbonization prior to steam activation results in a further increase in S_BET_ to about 269.1 m^2^/g. The change of contact time has been studied by Marques et al. [53] using steam activation at three different activation times of 1.21, 1.34 and 1.41 hours. They observed that the longer time yield the highest S_BET_. They attributed this to the fact that more activation time allowed the carbon to sufficiently, react with the activating agent, thereby, yielding better S_BET_.

Mohamed et al. [27] studied optimization of activation conditions employing a model relating textural properties and activation conditions in order to achieve the maximum surface area and micropore volume. He reported S_BET_ and micropore volume of 265 m^2^/g and 0.11 cm^3^/g when the sludge was activated at 838 °C and 80 min contact time. Other related studies conducted under similar activation conditions reported comparable values of obtained surface areas [42,53,54]. Compared to steam activation, CO_2_ proved promising in producing better textural properties of SBAC. However, CO_2_ exhibits lower reactivity in comparison with air and steam, because of its large molecule that restrict the complete diffusion in the porous carbonaceous structure [44]. Therefore, these studies revealed that activation is more effective at temperature higher than 800 °C.

A study conducted by Monsalvo et al. [9] investigated the effect of CO_2_ activation temperature and reaction time on the S_BET_ of SBAC. They found that compared to an increase in activation temperature from 700 °C to 800 °C , the increase in activation time from 0.5 h to 2 h significantly improved the S_BET_ from 20 m^2^/g to 94 m^2^/g at 800 °C. Further increase in activation time to 4 h results in less improvement in the S_BET_. The low CO_2_ activation rate arises as result of the hydrogen produced that adsorbed carbon actives sites and retard the activation rate [55]. Similarly as discussed above for steam activated SBAC, CO_2_ activation of two different types of sludge with (20% and 40% ash content) showed that the lower ash content SBAC yield the higher S_BET_ of 227.8 m^2^/g compared to high ash content activated carbon [53]. In order to enhance the S_BET_ of the physically activated sludge, washing with acid has been proven to be an effective approach. It was found that washing the char with HCl before activation dramatically increased the S_BET_ from 7 to 269 m^2^/g [45]. However, innovative techniques for physical activation still need further investigation [21]. Optimizing the physical activation operating conditions to produce activated carbon that has a maximum capacity for the uptake of pollutants, such as PC from water should be part of future investigations. Moreover, further improvement in the acid washing procedure is required to optimize the process of the physical activated carbon production from SS [21,45].

### 4.3. Chemically Activated SBAC

In this technique, a chemical reagent is employed for activation of the carbonized sludge char. The type of chemical reagents used and experimental conditions selected in this activation method play a crucial role in the production of SBAC. The characteristics (i.e., yield, S_BET_ and pore volume) of the produced SBAC depend mainly on four factors: reagent, reagent/sludge ratio, temperature and activation time [21]. Usually, optimum temperature is related to time of activation with high temperature requiring less time of activation. The most commonly used chemical reagents are ZnCl_2_, H_3_PO_4_, H_2_SO_4_, KOH and NaOH. However, a few other studies were found where SBAC was produced employing other chemicals reagents [56,57]. As shown in Table 4, the most effective amongst the aforementioned commonly used chemical reagents are KOH, NaOH, ZnCl_2_ and H_2_SO_4_. These studies were done using different pyrolysis, pre-treatment and post-treatment procedures. According to the best knowledge found in present literature, there is no systematic study that compares the efficiency of activating agents for production of SBAC. Also no published literature was found about optimizing yield, characteristics (i.e., S_BET_, pore volume and pore size distribution) of the SBAC in relation to uptake capacity of phenolic compounds. Table 4 shows the textural properties of SBAC produced using different chemical agents. The effectiveness of the most effective chemical agents (as well as other activation methods) vis-à-vis effects of production operating parameters on the characteristics of the produced SBAC are further discussed in details in the subsections below.

#### 4.3.1. ZnCl_2_ Activation

ZnCl_2_ has been known to be an effective activating reagent in the production of SBAC of better textural structure. Tay et al. [58] studied the effect of heating temperature, heating rate and ZnCl_2_ concentration on the pore distribution and S_BET_ of the final SBAC. Table 3 shows that for all ZnCl_2_ concentration the low heating temperature did not efficiently produce porous structure. Increased in temperature up to 600 °C yield an optimum surface structure with S_BET_ of 867 m^2^/g for 5 M ZnCl_2_. Increasing the temperature above 600 °C caused a decrease in S_BET_ due to sintering effect that damage the micropores, changing them to macroporous or mesoporous structures. Rozada et al. [51] investigated the ZnCl_2_, H_2_SO_4_ activation and carbonization of biological sludge. Compared to carbonized and H_2_SO_4_ SBAC as shown in Table 4, ZnCl_2_ activated SBAC showed the biggest value of S_BET_ (472 m^2^/g) and well developed macro porous structure but with small pore diameter. As per Table 4, the highest S_BET_ of ZnCl_2_-SBAC was 1092 m^2^/g with dominant mesoporous structure produced using paper mill sludge, at sludge to ZnCl_2_ ratio 3.5 [4]. Recently, Pirzadeh and Ghoreyshi [29] investigated the ZnCl_2_ activation of PMS at sludge to ZnCl_2_ ratio of 2:1.

The S_BET_ of the produced SBAC was relatively, low but with dominant micropore volume. In contrast, another study claimed that the ZnCl_2_ activation of PMS with high sludge to ZnCl_2_ ratio (0.9:1) generated an SBAC with S_BET_ of 907 m^2^/g and sufficient micropore structure [31].

#### 4.3.2. H_2_SO_4_ Activation

Previous studies suggest that H_2_SO_4_ was very effective in producing activated carbon with better textural properties. The highest S_BET_ achieved from H_2_SO_4_ activation of AWWTPS was 390 m^2^/g; produced by pyrolysis using H_2_SO_4_ at sludge ratio (1:1) for 30 min under temperature of 625 °C [59]. However, Martin et al. [24] and Salim Bousba [60], attained a low S_BET_ of 253 m^2^/g and 166.20 m^2^/g of H_2_SO_4_ activated SBAC respectively. They suggested that the low surface property was due to the high ash content of the precursor they used. Moreover, the resulting SBAC had high concentration of acidic groups which was attributed to the concentrated H_2_SO_4_ used for the activation. Rozada et al. [51] performed activation of anaerobic sludge using H_2_SO_4_ and ZnCl_2_. The resulting H_2_SO_4_ SBAC they produced exhibited lower S_BET_ (216 m^2^/g) compared to that produced using ZnCl_2_ activated (472 m^2^/g). As per the S_BET_, Table 4 suggests that H_2_SO_4_ is less effective than ZnCl_2_ in the production of SBAC.

#### 4.3.3. KOH/NaOH Activation

As shown in Table 4, KOH activated carbon attains the highest surface area of 1800 m^2^/g compared to activated carbon produced using ZnCl_2_ and H_2_SO_4_. Victor Manuel et al. [9] have investigated the effect of sludge to KOH ratio and heating temperature on the characteristics of the produced SBAC. The results showed that a positive impact of temperature and KOH to solid ratio. Significant improvement of S_BET_ was obtained from 131 to 950 m^2^/g and 262 m^2^/g to 1832 m^2^/g by increasing the activation temperature from 450 °C to 750 °C at solid to KOH ratio 1 and 3 respectively. Thus, increasing the sludge to KOH ratio from 1 to 3, at 750 °C activation temperature, results in almost 100% increase S_BET_ i.e., from 950 to 1832 m^2^/g. Similarly, activation using NaOH generates effective SBAC like other chemical agents. Zou et al. [44] produced SBAC possessing S_BET_ of 121.3 m^2^/g using NaOH using sludge to NaOH ratio of 1 at different heating temperatures (Table 4). The highest S_BET_ (up to 346 m^2^/g) and micro pore volume of the SBAC produced was attained at 600 °C activation temperature. Further increase in the activation temperature caused a decrease in S_BET_ which is associated to the degradation of porous structure and restricted porosity development. The well-developed porous structure of NaOH-activated SBAC was attributed to the oxidation of carbon into carbonate and intercalation of the produced Na-compounds during the reaction between carbon and NaOH [62].

#### 4.3.4. Other Activation Methods for SBAC Production

Beside the above traditional chemical methods, a recent literature review indicated that K_2_CO_3_ reagent [53,63,64], mixed Fenton reagent [61], electro-Fenton [65], microwave-assisted pyrolysis and activation [19,34,66,67,68,69], electrochemical method [57] and other emerging techniques [65,70,71,72] could also be employed for the production of SBAC. These techniques also have the potential to produce high S_BET_ SBAC characterized with high uptake capacity for organic and inorganic compounds. However, very few studies reportedly tested the adsorption of PC onto the SBAC produced via these activation methods. Among these methods, microwave-assisted chemical activation (MWCA) methods have gained more attention from researchers. This has been attributed to fact that such methods are fast and are characterized with uniform distribution of heat, high yield, improved mesoporous surface properties and cost savings over the conventional techniques [69,73].

Dos Reis et al. [68] compared the properties of GAC produced from SS using both conventional and MWCA using ZnCl_2_. Although both conventional and MWCA produced activated carbon of similar textural properties, the optimum S_BET_ of 501 m^2^/g was obtained using microwave at 980 W within 12 min activation time. In a recent study, Glaydson et al. [19], investigated the adsorption performance of removal of several PC (phenol, hydroquinone, *m*- and *o*-cresol, 2-chrorophenol, 2-nitrophenol) onto SBAC produced from municipal WWTP sludge using conventional and MWCA methods. The MWCA method produced SBAC of S_BET_ of 540 m^2^/g with high adsorption capacity for PC. Their study demonstrated very fast MWCA assisted SBAC production process with highest adsorption capacity of 1202.1 mg/g associated with hydroquione. Puchana-Roseroa et al. [69] prepared SBAC within 10 min under N_2_ atmosphere from tannery SS using a MWCA method with ZnCl_2_. The synthesized SBAC exhibited a surface area of 491.0 m^2^/g and good mesoporous surface properties. The author concluded that MWCA was a highly economical method for synthesis of SBAC compared to conventional heating as the production cost was nearly reduced by half [69].

The use of K_2_CO_3_ as an activating agent of SS was first reported by Marques et al. [53] from which they produced SBAC of S_BET_ 863.8 m^2^/g which served as effective adsorbent for phenol removal from water. Later, Cheng et al. [63] also reported the feasibility of activating WWTPS using K_2_CO_3_ to obtained SBAC having S_BET_ of up to 642 m^2^/g.

Recently, Gu et al. [66] produced a new iron-based magnetic SBAC (S_BET_ 341 m^2^/g) synthesized using sequential electro-Fenton (EF) activation and pyrolysis. The resulting SBAC was highly stable with superior physiochemical characteristics and the potential of yielding up to 80% of organic pollutant removal after three cycles of adsorption process. Frank et al. [70] employed hydrothermal carbonization (HTC) using MDSS of 75% water content without a prior thermal drying scheme. They reported that the water content of the MDSS served as a reaction medium during HTC at 180–250 °C [70]. However, their resulting SBAC S_BET_ of 109 m^2^ g^−1^ was low compared to even those produced by conventional methods. A novel composite SBAC of high S_BET_ (up to 641 m^2^/g) was prepared by dos Reis [71] via mixing sewage sludge with polysiloxanes and the mixture pyrolyzed at 500 or 600 °C under inert atmosphere of nitrogen. The produced composite SBAC exhibited high capacity for organic compounds adsorption [71]. Recently, Alvarez et al. [72] reported SBAC production using CO_2_ valorization in a fixed bed reactor at 800 °C which was preceded by two steps sequential washing, first using HCl, the followed by Na_2_CO_3_. Their new activation method involved very fast pyrolysis (within 15 min) which resulted in significantly improving the valorization perspectives of SS to obtain SBAC characterized by high presence of meso- and macropores and S_BET_ of 440 m^2^ g^−1^.

## 5. Adsorptive Characteristics of SBAC

The physiochemical nature of SBAC such as its pore structure, S_BET_ and its surface chemistry (presence of oxygen functionalities on its surface) are the key characteristics that influence the adsorption capacity and also control the mechanism of PC adsorption on SBAC interface [26,74,75].

### 5.1. Pore Structure of SBAC

Based on the pore structure of SBAC one can determine the accessibility of PC on the SBAC internal surface. For instance, phenol which is a relatively small sized molecule can easily penetrate into microporous structures, normal organic compounds (NOMs) can enter mesoporous structures while bacteria can assess macropores. Khalili et al. [4] demonstrated that the SBAC produced from PMS exhibited better adsorption and strong affinity for phenols compared to a commercial GAC. They associated this behavior to the mesoporous structure of PMS SBAC which resulted in improved diffusion-adsorption of phenol molecules in the SBAC inner surface. Martin et al. [24] also found that the low adsorption capacity of SBAC was due to the development of less microspore structures as PC adsorbed in the pores size having diameter greater than 1 nm [76]. In addition, the pore size also influences the kinetics of adsorption onto SBAC [77]. Daojing et al. [28] studied the behavior of SBAC porosity on the adsorption of some PC. The authors revealed that the SBAC having a mesoporous structure has a faster diffusion rate for the PC compared to SBAC with a microporous structure. They attributed this phenomenon to the molecular sizes of the PC which were slightly higher than the micropore diameter. Consequently, the penetration of PC was faster in mesoporous SBAC [28].

### 5.2. Functional Groups on the Surface of SBAC

Apart from the pore structure of SBAC, the adsorption capacity and mechanism of SBAC is significantly influenced by its chemical characteristics, i.e., the presence of oxygen functional groups on its surface [23]. SBAC usually exhibit excellent adsorption performances owing to high S_BET_ and percentage of micropores combined with hydrophilic high-activity oxygen-containing functional groups of OH, NH_2,_ NO_2_, C-O, O-C-O and C=O [23]. These functional groups may be acidic, basic or neutral in nature [78]. The type of functional group present on the surface of SBAC determines the surface charge, hydrophobicity and the electronic density of graphene layers [23,79]. Oxygen functionalities on the surface of SBAC are present in various forms such as carboxyl, carbonyl, hydroxyl, pyrene, quinone, etc. [23,78]. These functionalities can be developed as result of the different chemical and physical activation methods discussed earlier. Based on data reported in previous studies, the presence of acidic surface oxygen functional groups result in a decrease in uptake of PC whereas the increase in the basicity of SBAC surface play a positive effect on the adsorption capacity of PC [80]. Moreover, a decrease in the acidity of the SBAC surface also improves the adsorption capacity of PC on SBAC [81,82]. Yin et al. [83] noticed that the behavior of adsorption of phenol on SBAC was not only associated to the physisorption but also to the oxidative polymerization reactions between phenol and the carbon surface [84]. Therefore, the presence of oxygen functional groups played a dominant role and promotes adsorption. Mansalvo et al. [9] studied the adsorption performance of 4-chlorophenol on air-, CO_2_- and KOH-activated carbons. The authors discovered that the nitrogen and sulphur substituents groups were higher in air-GAC compared to CO_2_ and KOH-GAC. The adsorption behavior governed π-π interactions between the aromatic ring of 4-CP and the surface oxygen, nitrogen and sulphur groups on the AC. Therefore, air-GAC, even with low surface area, showed better affinity for 4-CP. The KOH-GAC exhibited higher adsorption capacity which was due to its well-developed porosity structure. Masomia et al. [31] investigated the behavior of adsorption mechanism of 4-nitrophenol and 2-chlorophenol on PMS based SBAC. Their results proposed that the mechanism was governed by π-π interactions between hydroxyl groups and nitro groups on the SBAC surface.

## 6. Adsorption of Phenolic Compounds on SBAC

The characteristics of PC in relation to their influence on adsorption on different SBAC are discussed in the subheadings below.

### 6.1. Adsorptive Characteristics of Phenolic Compounds

The mechanism and adsorption capacity of PC on the surface of SBAC are also influenced by the characteristics of the adsorbate. These characteristics include: (i) molecular size, which defines the accessibility into the pores of SBAC and kinetics of adsorption; (ii) solubility which controls the hydrophobic interactions; (iii) its pKa value which governs the dissociation properties of the adsorbate (if an electrolyte) in the solution [85]. In addition the presence of substituent groups such as nitro and chloro are indirectly involved in adsorption by alteration of molecular properties of adsorbate. Previous studies indicated that the interaction between phenol and SBAC surface can be increased by the introduction of electron-withdrawing groups. Both nitro and chloro groups exhibited strong electron-withdrawing ability and thus the presence of these groups in PC such as nitrophenols and chlorophenols showed better and stronger affinity to get attracted to the SBAC surface [61,86]. The time for attainment of equilibrium was found to follow the order phenol > 2-chlorophenol > 4-nitrophenol, while the adsorption capacity increases according to the order: 4-nitro- phenol > 2 chlorophenol > phenol [19,31]. The equilibrium time was controlled by the molecular size of the adsorbate, i.e., the bigger the size the longer is the diffusion time whereas adsorption capacity was associated to the substituent groups on the adsorbate that enhanced the interaction of phenols with the SBAC surface.

### 6.2. Adsorption of Phenolic Compounds on Dried Sludge

**Figure 2:**
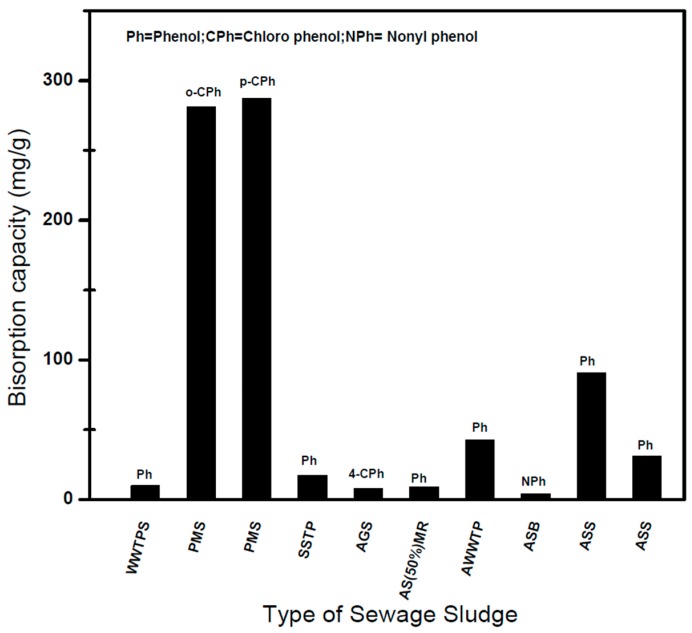
Dried sludge uptake capacity for phenolic compounds.

Adsorption using dried sludge obtained from different WWTP is also an effective means of removing PC from wastewater streams. Temperature and pH are the two critical parameters having great impact on the efficiency of adsorption of PC using dried sludge [87,88]. The pH of the solution significantly influences the adsorption process as it affects the degree of speciation of the PC as well as the surface properties of sludge. The isoelectric point of an anaerobically dried sludge biomass is usually between 1 and 3 [89]. Therefore at low pH, the entire surface charge on the sludge cell will become positive and at pH values above 8, the surface charge will change to negative, leading to an increase and decrease in the binding affinity of the SBAC towards phenolic compounds, respectively [90]. Additionally, it is also well known that the speciation of the phenol is greatly influenced by its inherent pKa of 9.89 whereby it could either deprotonate to phenolate ions (i.e., phenoxy radicals) or form phenolate ions depending on whether the solution pH is acidic or basic, respectively [91]. Figure 2 and Table 5 compare the sorption capacity of PC using different dried sludges at optimum pH and temperature.

The highest sorption was observed to be associated with paper mill sludge during uptake of *o*-chlorophenol (281 mg/g) and *p*-chlorophenol (287.2 mg/g) at pH 1 and temperature 25 °C [92]. At low pH the surface of biosorbent become dominant with hydronium ion which boost the binding capacity and thus increased chlorinated phenol interaction due to strong attractive forces [93]. Aksu and Yener [94] investigated the adsorption of phenol using dried sludge and the highest uptake capacity of phenol was obtained at pH 1.0 and temperature 25 °C was 91 mg/g. In another study Akzu and Akpinar [95] studied the adsorption of phenol using dried aerobic activated sludge. The optimum pH obtained was 1, with a maximum phenol adsorption capacity of 180.9 mg/g.

However, Moura et al. [96] utilized WWTP sludge for the removal of phenol and found a very low adsorption capacity of 0.06 mg/g at pH 7. This was associated to low interaction of the binding sites of the sludge with phenol molecules. Arslan and Dursun [97] have studied the adsorption of phenol using dried sludge and found that loading capacity increased by increasing the temperature and pH and the maximum adsorption capacity achieved was 42.7 mg/g at pH 8 and a temperature of 40 °C (Table 4).

The increase in uptake due to an increase in pH was attributed to speciation of phenols, alteration in sludge surface chemistry, and improved strong electrostatic force interactions between phenols and sludge [102,103].

However, above pH 8 the biosorption capacity decreased due to electrostatic repulsion of the negatively charge surface of sludge. Gao and Wang [91] and Sulaymon et al. [98] investigated the biosorption capacity of anaerobic sludge for phenolic compound removal. Gao and Wang [90] found that the sorption was exothermic in nature and the maximum biosorption of *o*-chlorophenol was achieved at low pH value, i.e., due to the increase in binding capacity of sludge surface and OH- and Cl- of chlorophenol. Sulaymon et al. [98] reported the maximum adsorption capacity of phenol was 90.54 mg/g (Table 4) at 30 °C. The adsorption of phenol is governed by the reactions between the functional group of phenol with carboxylic, amine and amide groups present on the surface of the anaerobic sludge. Nonetheless, the presence of other ions affects the adsorption of phenol due to the competitive environment created for the dried sludge active sites. Thawornchaisit and Pakulanon [99] and Sulaymon et al. [98] investigated the effect of the presence of Cu and Pb on the biosorption of phenol. Both studies reported that the biosorption of phenol using dried sludge decreased due to the presence of Cu and Pb ions, respectively. They suggested that the small size of Cu and Pb ions compared to the larger phenol molecules led to the heavy metals’ higher attraction onto the binding sites of the sludge.

### 6.3. Adsorption of Phenolic Compounds on Physically Activated SBAC

**Figure 3:**
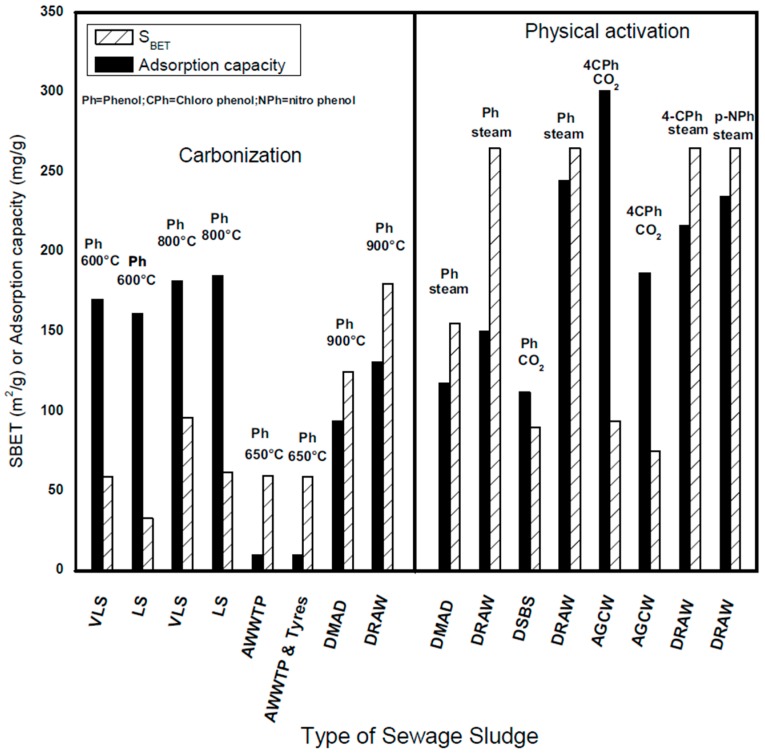
Achieved SBET in relation to uptake for phenolic compounds for physically activted SBAC.

The efficiency of SBAC for the removal of PC in water is mainly influenced by the textural properties (S_BET_ and micro pore volume) and surface chemistry (functional groups) on the SBAC surface [104,105]. Figure 3 depicts the comparison of adsorption capacity of PC of some SBACs produced using different physical methods. The figure indicates that the low S_BET_ carbonaceous carbon produced at 600 °C using VL and VLS showed a good uptake capacity of phenol of 170 mg/g and 161 mg/g respectively [25].

The high uptake capacity of phenol at low S_BET_ demonstrates that the adsorption is governed by the surface chemistry of the carbonaceous carbon [106] i.e., the formation of functional groups on the surface of carbonaceous carbon was responsible for the elimination of phenol. The adsorption capacity is found to be further increased due to the high S_BET_ obtained as a result of increasing the LS and VLS carbonization temperature to 1000 °C and 800 °C. A similar tendency in seen in carbonaceous SBAC produced using anaerobic sludge and a mixture of tyres and sludge with S_BET_ of 60 m^2^/g and 59 m^2^/g and dominant mesoporous structures [51]. However, in this case, the adsorption capacity of phenol was found very low, about 9.8 mg/g and 10.1 mg/g, respectively. The authors claimed that the mesoporous structure and low reactivity of the surface of the carbonaceous carbons results in low uptake of phenols. This was improved by chemical activation using H_2_SO_4_ (as discussed earlier). Also as mentioned earlier, physical activated SS particularly, using steam, exhibited higher S_BET_ compared to carbonization [53]. Thus, the adsorption capacity of steam-activated SBAC is usually higher in comparison with those activated via carbonization (Figure 2). It is apparent that the steam activated carbons don’t only exhibit enhanced textural properties but also produce surface functional groups that result in improved PC adsorptive capacity. For example, the S_BET_ and adsorption capacity of carbonized DSS increased from 180 m^2^/g to 265 m^2^/g and 131 mg/g to 150 mg/g when further activated using steam, respectively [42]. Recent studies have claimed a similar behavior [20,22]. The optimized operating condition of steam activation investigated on DSS generate a high surface area and dominant micropore volume that results in a highest adsorption capacity of 244 m^2^/g for phenol (Figure 2) [27]. CO_2_ activated SS, exhibited better S_BET_ yielding good adsorption capacity for PC. Monsalvo et al. [9] observed a slight increase in S_BET_ from 75 m^2^/g to 94 m^2^/g when the activation temperature was raised from 700 to 800 °C with no significant improvement in the uptake capacity of chlorophenol. However, increasing the contact time from 2 h to 4 h resulted in an increase in the adsorption capacity from 241 mg/g to 301 mg/g. In a similar study, the adsorption capacity of air-activated SBAC was found to be associated with the S_BET_ (Figure 3).

### 6.4. Adsorption of Phenolic Compounds on Chemically Activated SBAC

**Figure 4:**
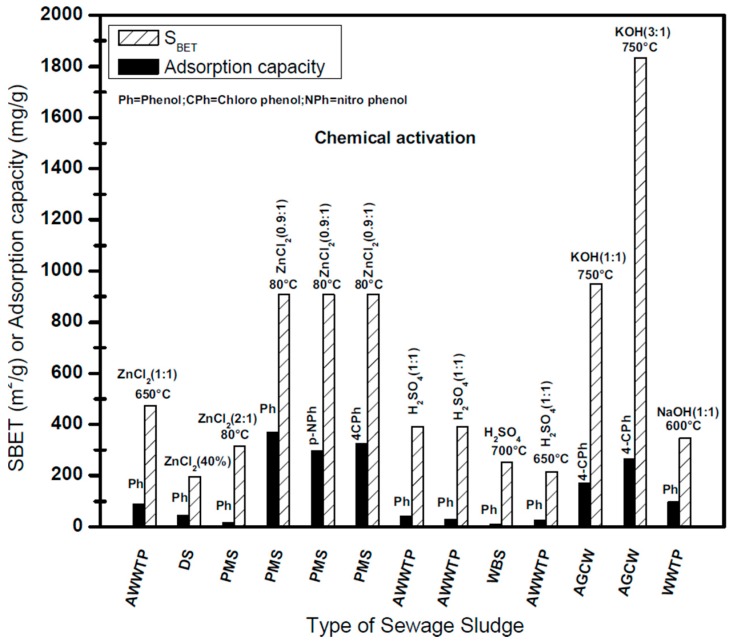
Achieved SBET in relation to uptake for phenolic compounds for chemically activted SBAC.

Reported adsorption capacities for the sorption of some PC onto SBAC produced using chemical activation techniques are shown in Figure 4.

The highest adsorption capacity of 265.08 mg/g was associated with 4-chlorophenol and was exhibited by KOH-activated SBAC at a KOH to sludge ratio 3:1 [9]. The uptake capacity dropped to 170 mg/g when the KOH to sludge ratio was reduced to 1:1. The high adsorption capacity was due to the development of high porosity with a large surface of 1832 m^2^/g and 950 m^2^/g, respectively. Hence, the significant improvement of the S_BET_ when the sludge to KOH ratio was increased from 1 to 3 didn’t yield higher adsorption capacity for 4-chlorophenol as expected. This could be attributed to the loss of S-content and N-content when activated at 750 °C. Like other activating agents, activation using NaOH showed better adsorption capacity for phenol uptake on SBAC. Figure 4 shows that despite the low S_BET_ of 346 m^2^/g (due to the inorganic content on the precursor) NaOH-activated carbon exhibited better adsorption capacity for phenol [44]. The presence of high content of oxygen-containing functional groups on the surface of NaOH-activated carbon results in increased electrostatic interactions and thus high binding with phenols [107,108]. In the case of ZnCl_2_ activated carbon, 370 mg/g was the highest adsorption capacity of phenol obtained by using paper mill sludge with low ZnCl_2_ to sludge ratio 0.9:1 [31]. The well-developed porous structure of activated carbon and the presence of hydroxyl (OH) groups on its surface leads to the enhanced interaction with phenol molecules. In a previous study [58] using ADDWWTP with low ZnCl_2_ to sludge ratio a very low adsorption capacity of phenol was exhibited, though the produced SBAC had a high BET surface area of 867.61 m^2^/g. This could be the result of two major reasons: (i) the BET surface was analyzed using nitrogen molecules which are small in size and enter into the pores that were not large enough for large molecules like phenol and (ii) the most important reason is the surface chemistry of activated carbon, particularly the presence of oxygen-containing functional groups that directly reflect the adsorption of phenol. Otero et al. [59] investigated the adsorption of phenol using H_2_SO_4_-activated carbons of two different diameters (0.12 and 0.5 nm). Activated carbon of lower diameter (0.12 nm) revealed about 70% greater adsorption of phenol compared to 0.5 nm activated carbon. As discussed above, SBAC produced using H_2_SO_4_ activation was found to possess low BET surface and thus, exhibited low uptake of phenol molecules.

### 6.5. Effect of Operation Conditions

As a result of the dominant role they play, initial pH, temperature, contact time and adsorbent dose are the most investigated operating conditions employed to evaluate the adsorptive performance of PC on SBAC. Initial pH of the solution is the key experimental parameter that has a a very profound impact on the interaction of PC with GAC. This is because the pH greatly controls the physiochemical interactions between adsorbent and adsorbate in solution. For instance, when the pH is greater than the point of zero charge pH (i.e., pH_pzc_) the surface of carbon is positive in nature and as pH > pH_pzc_ the carbon surface changed to negative. Similarly, at pH < pKa, the phenols exist in protonated form, while at pH > pKa they predominantly dissociate to phenolate ions [91]. The change in surface charge of carbon and PC dissociation due to pH of the solution changes results in either increase or decrease in electrostatic interaction between them. Thus, an increase in the pH of solution is susceptible to cause the transformation of the negatively charged carbon surface and deprotonation of PC [20]. This may result in an increase in the electrostatic repulsion between adsorbed PC molecules with the negative charge on the SBAC surface. This leads to a reduction in adsorption capacity. However, at low pH values, the positively charged surface of SBAC develops a stronger interface with protonated PC and leads to improved uptake of phenols on the SBAC surface.

Seda et al. [97] studied the effect of pH on the biosorption of PC on dried sludge. The authors concluded from the results that by increasing the pH of the solution up to pH 8, the uptake of PC was increased. Above pH 8, the adsorption of PC on dried sludge started to decrease. The phenomenon of high uptake of phenol at low pH is attributed to the positive charge on the binding sites of the dried sludge. However, the low phenol uptake at pH > 8 is associated to the formation of negative phenolate ions and a negatively charged surface of the dried sludge that enhances the electrostatic repulsion forces and thus low adsorption capacity of phenol on dried sludge was found. Also, this behavior could be ascribed to the presence of OH^−^ ions at high pH values that covers the adsorption sites of dried sludge and restricts the access of phenol molecules on dried sludge surface [109]. Salim et al. [60] studied the effect of pH on the removal of phenol using SBAC. The maximum removal of about 80% was found at pH 6–8. A further increase in pH caused a rapid decrease in phenol removal. The mechanism is associated to the electrostatic repulsion between the negatively charge surface of the SBAC and ionic phenols in solution. At pH 12, the maximum removal was around 21% which indicates a chemisorption adsorption mechanism. Thus, according to previous studies, we can conclude the maximum removal of phenols on SBAC can be achieved in the pH 6–8 range.

Adsorption temperature is another important experimental parameter that has a very critical effect on the mechanism and uptake capacity of phenols on SBAC. A number of authors have conducted studies to understand how the temperature affects the adsorption capacity of PC on SBAC. Gao and Wang [90] investigated the removal efficiency of 4-CP and 2, 4-DCP on anaerobic SS based SBAC. The results showed that the adsorption of these PC decreased with increasing temperature, suggesting an exothermic adsorption behavior. Similar behavior was also reported by Mansalvo et al. [39] and Masomi et al. [31] for the adsorption of 4-CP and phenol on SBAC, respectively. These authors attributed this phenomenon to the increase in temperature initiating a weakening of attraction of phenol molecules with the carbon surface and thus allowing the adsorbed phenol molecules to escape from the adsorbed phase to the bulk phase. In contrast, for biosorption of phenols on dried activated sludge, Seda et al. [98], noticed that the adsorption capacity of phenols increased with increasing temperature from 10 °C to 40 °C. In this case, the sorption mechanism of phenol on dried activated sludge was endothermic in nature. They attributed that to a rupture or breakage of bonds caused by the temperature increase [95,110]. This resulted in the formation of new active binding sites.

## 7. Isotherms and Mechanisms of Phenolic Compound Adsorption on SBAC

Assessment of the mechanisms of SBAC adsorption is usually conducted using equilibrium and kinetics approaches in batch shake-flask experiments from which adsorption isotherms could be developed in order to obtain the SBAC maximum uptake capacity. Meanwhile, fixed-bed column experiments (continuous process) are conducted to produce breakthrough curves representing changes in adsorbate concentration with time under selected conditions. Practical industrial and large scale water and wastewater GAC treatment application processes are based on the continuous process hence, rendering the fixed bed study more beneficial. However, very few studies have investigated the adsorption behavior of PC on SBAC using continuous modes for breakthrough curve analysis [100]. The adsorption mechanisms of PC on SBACs are discussed in details in the sections below.

### 7.1. Adsorption Isotherms

Adsorption isotherms models are investigated to articulate the distribution behaviors of PC between the SBAC surface and the water phase [111]. The most common isotherm models reported that satisfactorily correlate the uptake of PC on SBAC and their equilibrium concentrations in solution are the Freundlich and Langmuir models as summarized in Table 6. The Freundlich model given in Equation (1) is based on the assumption that adsorption occur in a heterogeneous system at different active sites and energies. Also, it assumes a multilayer adsorption of adsorbate [112]:

(1) $$\ln q_{e}=\ln K_{f}+\frac{1}{n}\ln C_{e}$$

where, *q_e_* is the adsorption capacity (mg/g) at equilibrium; *C_e_* is the adsorption concentration (mg/g) at equilibrium; *K_f_* is the Freundlich constant which indicates the adsorption capacity for an adsorbate; 1/*n* is the heterogeneity factor indicating the percentage of heterogeneity of activated carbon surface and the affinity of adsorption sites with the adsorbate molecules. Ten years later in 1916, Langmuir proposed a new model that describes monolayer adsorption onto homogenous active sites with identical energy levels [113]. The Langmuir model equation is given as follows:

(2) $$\frac{C_{e}}{q_{e}}=\frac{1}{b}q_{m}+\frac{C_{e}}{q_{m}}\;a\;=\;1$$

where *q_m_* is monolayer adsorption capacity (mg/g); *b* is Langmuir isotherm constant. The dimensionless feature of Langmuir (*R_L_*) is used to evaluate the feasibility of model expressed as:

(3) $$R_{L}=\frac{1}{1+bC_{o}}$$

where *b* is the Langmuir constant and *C_o_* is the initial concentration. *R_L_* describes the shape of the Langmuir isotherm. If *R_L_* > 0 (unfavorable), *R_L_* = 1 (irreversible), *R_L_* = 0 or 0 < *R_L_* > 1 (favorable). Nevertheless, other models such the Sips model [114] and Redlich and Peterson [115] were developed later to complement behaviors that couldn’t be explained by the earlier two models. The Sip model is a combined form of the Langmuir and Freundlich models that predicts the behavior of adsorbed molecules as per the theory of Sip which was based on the assumption that the molecules of solute (adsorbate) may adhere to more than one active site [114]. Accordingly, the Sip models act as the Langmuir one, i.e., monolayer adsorption at high concentration while at low concentration it changes to a Freundlich model, i.e., multilayer adsorption. The Sip model is given as follows:

(4) $$q_{e}\;=\;\frac{n_{\mathrm{mLF}}{{(K}_{\mathrm{LF}}C_{e})}^{1/\mathrm{nLF}}}{{(1\;+\;(K}_{\mathrm{LF}}C_{e}{)}^{1/\mathrm{nLF}})}$$

where n is the adsorbed amount at equilibrium, n_mLF_ the Langmuir-Freundlich maximum adsorption capacity (mg/g), K_LF_ is heterogeneity equilibrium constant, and nLF is the heterogeneity parameter, lying between 0 and 1.

In 1959, Redlich and Peterson proposed another model written as Equation (5) which consists of three adsorption parameters [115]. The model produces satisfactory results in most of adsorption systems where the Langmuir and Freundlich models fail:

(5) $$q_{e}\;=\;\frac{K_{R}C_{e}\;}{{1+a}_{r}C_{e}\beta }$$

where *K_R_* is the R-P isotherm constant (L/mg), a_r_ is also a constant (L/mg)^1/β^ and β is the exponent between 0 to 1. This isotherm has an exponential dependence on the concentration (in the denominator) and a linear relation with the concentration (in the nominator). The equation approaches a Langmuir model at low concentration and describes a Freundlich model at high concentration. This can be applied to both homogenous and heterogeneous systems [116].

Table 6 shows the comparison of isotherms parameters of Langmuir and Freundlich for adsorption of PC on SBAC. A preliminary indication of the adsorption capacity of a GAC for any compound can be estimated based on the S_BET_. However, many researchers have found that there is no linear relation of S_BET_ of activated carbon with its adsorption capacity. For instance, as shown in Table 6, the KOH-activated carbon of highest surface area of 1832 m^2^/g showed an adsorption capacity for 4-chlorophenol of 265.8 mg/g. However the same sludge when activated using CO_2_ and air exhibited a low surface area of 94 m^2^/g and 91 m^2^/g (20 times lower than KOH-activated carbon) but had a high adsorption capacity of 301 mg/g and 223 mg/g, respectively [9]. The adsorption was associated to higher π-π interactions of 4-chlorophenol with the activated carbon surface. As explained earlier, the presence of nitrogen, oxygen and sulfur surface functional groups, to a large extent influences the π-π interaction and leads to a strong adsorption behavior [19,117]. Particularly, S and N groups on the surface of carbon reflect increased affinity of activated carbon toward 4-chlorophenol [118]. The high temperature activation of KOH caused a significant loss of N-content and thus decreases its adsorption capacity. Otero et al. [59] produced SBAC with a surface area of 390 m^2^/g using the H_2_SO_4_ activation method. The maximum adsorption capacity for phenol obtained from the Langmuir model was 42.04 mg/g. A similar range of surface area of SBAC of 346 m^2^/g was produced by Zou et al. [44] using the NaOH activation method. The adsorption capacity of the NaOH-activated SBAC was almost double (i.e., 96.5 mg/g) compared to H_2_SO_4_ activated carbon. This could be explained by the mechanism of adsorption of phenol on the surface of activated carbon which is associated to surface electrostatic interactions. The presence of a large number of oxygen functional groups on the surface of NaOH-activated carbon compared to H_2_SO_4_-activated SBAC is susceptible to provoke an increase in phenol adsorption.

As given in Table 6, different SBACs show different orders of adsorption capacity for various PC. The hydrophobic nature of SBAC suggests a strong affinity towards organic compounds with limited solubility in water, i.e., hydrophobic compounds have higher adsorptions than hydrophilic compounds [119]. For example, using the Langmuir model, Jain et al. [120] found that SBAC gave an increasing order of adsorption capacity as the number of chloro groups increases (Table 6). Hence, this suggests a solubility factor effect of phenols in aqueous solution. An increase in the chloro groups in a phenol limits its solubility in water and thus increases its adsorption capacity. Similar behavior was also observed by Bousba and Abdeslam [30] for a H_2_SO_4_-activated SBAC which exhibited double the adsorption capacity for 2-chlorophenol compared to phenol. However, Masomiet al. [31] and Mohamed et al. [27] observed that the affinity of SBAC was higher for phenol compared to chloro-phenol and even far less for nonylphenol, a compound having the least solubility compared to chloro-phenol and phenol. Therefore, in this case the solubility factor appears secondary and the binding of phenols on the surface of a SBAC was attributed to the dispersion effect and the mechanism of adsorption is the ionic interaction of surface basic groups (electron-donors) with phenol molecules (electron-acceptors).

### 7.2. Mechanisms of Adsorption of Phenolic Compounds on SBAC

The mechanisms of adsorption of PC on SBACs have been extensively studied in the last decade [20,39,101,121,122,123]. However due to its complexity, the mechanism is not yet understood well. Nevertheless it is known that the behavior of adsorption of PC on SBACs mainly depends on the following key factors:

- Characteristics of the SBAC. These include the pore size distribution (surface area, pore volume), presence of oxygen functionalities on the carbon surface, ash content and others like mineral content.
- Characteristics of the adsorbate. These include the molecular size of the adsorbate, its pKa value, functional groups and polarity.
- Experimental conditions. These include the pH of the solution, temperature, ionic nature and concentration of the solution.

Based on the influence of the abovementioned factors, the behavior of a SBAC may be associated to two proposed mechanisms; electron-donor acceptor complex and π-π dispersive interaction [122,123]. The π-π interaction mechanism was first proposed by Mattson et al. [124]. It demonstrates that the adsorption is due to π-π-interactions between the the aromatic ring graphene layer of the SBAC and the aromatic ring of the PC. The presence of acidic functional groups on the SBAC surface leads to weaker interactions with PC due to the removal of formation of positive sides in the p-band system of the graphite basal planes [125]. Mattson et al. [124] were the first to propose adsorption of PC on SBAC by donor-acceptor complex behavior. They proposed that the carbonyl oxygen functional groups on the surface of SBACs act as an electron donor while the aromatic rings of PC behave as an acceptor. The aromatic rings further form donor acceptor complexes with rings of the basal system when the carbonyl groups become saturated. This theory demonstrates that the the oxidation of SBACs changes the carbonyl groups (C=O) to carboxyl groups (COOH) and therefore results in a decrease in the PC uptake. Even though the mechanisms proposed above could provide adequate explanations, however, they cannot be applicable under all scenarios. Thus, apart from above two mechanisms other various physiochemical factors such as hydrogen bonding, ion exchange, covalent bonding, and Van der Waals forces can be associated to the adsorption of PC on SBAC.

## 8. Regeneration of Spent SBAC

For broader and more practical applications of GAC in industrial water treatment, the regeneration potential of spent GACs is one of the important characteristics to be considered for the selection of GACs. This is due to the fact that economical regeneration after exhaustion would bring significant cost savings of up to 20–40% reduction in the cost of virgin GAC [22]. Additionally, it helps in recovering of adsorbate and also to understand the likely mechanism of the adsorbate adsorption process [10]. The efficiency of the spent SBAC regeneration process would depend on the characteristics of the SBAC utilized vis-à-vis the mechanism of adsorption of PC onto such SBACs (physisorption or chemisorption as discussed in the previous sections). The three types of regeneration methods that were mainly employed for spent GACs, which include, thermal, chemical, and electrochemical regeneration [126,127,128], are discussed below.

### 8.1. Thermal Regeneration of Spent SBAC

Thermal regeneration involves the treatment of spent GAC in the absence of oxygen at around 300 °C to 850 °C. This allows the vitalization and desorption of physiosorbed adsorbate without oxidation of the spent GAC. Thermal regeneration is capable of regeneration of at least 90% of spent GACs [129]. However, the regeneration process could have negative effects on the characteristics of the recovered GACs, i.e., changes in the pore size distribution [130], loss of carbon content and high cost due to energy consumption. Moreover, one of the challenges of thermal regeneration is the formation of undesirable light gaseous and polymeric products from chemisorbed pollutants when heated at high temperature [122]. This causes hindrance in the desorption process which negatively affects the adsorption capacity of the regenerated SBAC [122].

Recently, Gupta and Garg [131] thermally regenerated spent SBAC at 700 °C for 1 h and they used the restored product for phenol adsorption. They found that the thermal regeneration led to a reduction in the BET surface area by 53–60% with a considerable reduction in the total pore volume. This resulted in a significant drop in the adsorption capacity by 55–65% of that of the original SBAC. With three consecutive cycles of thermal regeneration by heating at 300 °C for 30 min, Cheng et al. [63] reported almost complete restoration of the SBAC after the first cycle, and a slight decrease after consecutive regeneration cycles. Under similar regeneration conditions to those of Cheng et al. [63] and three adsorption–regeneration cycles, Li et al. [111], effectively regenerated SBAC used for the removal of some organic compounds. They reported also a high regeneration efficiency of the SBAC they used which exhibited a slight decline in the performance.

Currently, regeneration studies for SBAC employed for adsorption of PC are very scant. It has been reported that the adsorption capacity of PC on GAC was found to decrease with increasing regeneration cycles for thermally regenerated activated carbon [132]. According to Monreno-Castilla et al. [133], nitrophenol and *p*-cresol have better interaction and strong adsorption compared to phenol on the GAC surface. This results in a substantial reduction in adsorption capacities from 210 mg/g to 30 mg/g and 200 mg/g to 70 mg/g for N-Ph and *m*-aminophenol after the third regeneration cycle, respectively. Thus, the strongly bonded chemisorbed PC on GACs cause the adsorption capacity to decrease with successive regeneration cycles.

### 8.2. Chemical Regeneration of Spent SBAC

Chemical regeneration involves the utilization of suitable chemical reagents on exhausted SBAC. Several studies have investigated the potential of different chemical reagents for the regeneration efficiency of spent or exhausted SBAC after water treatment [134,135,136]. The literature indicates that the most commonly used chemical agents for regeneration of used SBACs include electrolyte solutions: NaCl and NaNO_3_; acids: H_3_PO_4_, H_2_SO_4_, HCl, HNO_3_; base: NaOH [10] and organic compounds: ethanol, methanol, acetone, benzene and ethanol/acetic acid [137]. However, no study was found that focused on the potential of using these chemicals for regeneration of SBAC used for PC adsorption. Based on studies of other classes of organic compounds, it is expected that efficiency of chemically regenerated exhausted SBAC would be governed by solvent interactions with the internal micropores of the SBAC and with the PC. The better the interaction of reagents with PC and carbon surface the higher the regeneration efficiency. Ferro-Garcia et al. [136] reported the percentage of *o*-chlorophenol and *m*-chlorophenol extracted by using different reagents from exhausted GAC. They found that ethanol was more effective in extracting the phenolic compound compared to other reagents. Similar behavior was also seen in other studies [138]. In addition the removal percentage of *m*-chlorophenol is lower compared to *o*-chlorophenol for all solvents.

### 8.3. Electrochemical Regeneration of Spent SBAC

Similarly, as in the case of the other regeneration methods, no documented research was found on electrochemical regeneration of SBACs directly related to PC. Available literature on this subject suggests that electrochemical regeneration is more effective compared to thermal regeneration. This regeneration process can recover over 95% of the uptake capacity for fresh GAC used for adsorption of phenols and other organic compounds [139,140]. Generally, compared to thermal methods, electrochemical regeneration showed high efficiencies, low loss of carbon content, and fast desorption of PC from the carbon surface through oxidation and also suitability at a low scale [141]. However, some noted disadvantages associated with the electrochemical techniques include high energy consumption, contamination of electrodes, and surface amendment due to the high current [142]. Time and current are the two most important parameters which control the efficiency of electrochemical regeneration. Longer desorption time yields higher regeneration efficiency, though at the expense of an increase in the energy consumption required for the process [142]. Similarly, an increase in the electric current also results in an increase in the desorption efficiency of regeneration. However, high current may alter the surface structure of the GAC. Therefore, it is necessary to determine the optimum regeneration current and time for better regenerated GAC [128].

## 9. Sustainable SBAC Production and Utilization for PC Removal from Water

In the context of sustainability, SBAC cost-effectiveness is expected to be a function of the type of SS, the costs of SS processing (dewatering, drying, etc.), SBAC production, regeneration and final disposal in relation to the produced SBAC effectiveness towards PC adsorption over its active lifetime [10]. Thus, as result of a number of influencing parameters, the economic evaluation of SBAC utilization for PC removal from water is expected to be better evaluated using LCA. LCA as a holistic decision-making tool would incorporate all the involving processes: SBAC production, utilization for PC removal from water and disposal method considering wide ranges of available alternative options/scenarios vis-à-vis the target PC decontamination effectiveness, associated environmental impacts and cost implications. Hence, the interdependence of different components of the processes would be accounted for, thereby enabling better and accurate optimization as well as selection of the best processes combinations to ensure overall cost-effectiveness and environmental sustainability. However, LCA of the utilization of SBAC for the removal of pollutants from water are rarely found in the literature [10,143].

## 10. Conclusions

Rapid population growth coupled with urbanization and industrialization has led to stricter legislations that put pressure on adequate management of the huge quantities of sewage sludge produced by wastewater treatment plants (WWTPs). For sustainable development, the need to seek a cost-effective, environmentally sound strategy for sludge management couldn’t be overemphasized. For their known detrimental health and environmental impact, removal of these compounds from wastewater streams to meet regulatory discharge standards becomes mandatory. Hence, this paper focuses on the current literature on the removal of PC from aqueous systems using adsorption on SBACs. Based on the review, the following conclusions and recommendations for future investigations are drawn:

- Different SBACs exhibiting divergent physicochemical characteristics as well as adsorption performances for removal of PC from water were attributed to the diverse sources of SS as well as activation techniques employed for SBAC production.
- Although chemical activation techniques produce better SBAC textural properties and superior PC adsorptive performance compared to physical activation, t more research works are needed to harness the advances in material science to improve the functional groups and textural properties of SBACs as well as the low performance of physical activation methods.
- Investigation of new and novel chemical activation reagents and combined chemical and physical activation systems are rare. Thus, these need to be explored for producing better SBACs for improved effectiveness of PC removal from water.
- The Freundlich and Langmuir models were the most satisfactorily isotherm models that describe well the uptake of PC on both dried and activated SBAC.
- Even though practical industrial and large scale applications of water and wastewater GAC treatment processes are based on the continuous process hence rendering fixed bed studies more beneficial. However, very few studies have investigated the adsorption behavior of PC on SBACs using continuous modes for breakthrough curve analysis.
- Most of the investigated PC mainly included parent the phenol molecule and simple derivatives like chlorophenols, bromophenols and nitrophenols. Thus, the adsorption performances of SBACs for the removal of toxic compounds such as catechol, resorcinol, benzoquinone and several other PC of environmental significant in single and multi-systems are also important to be evaluated.
- Despite the established economic benefits of regeneration of spent SBACs using different techniques, studies that evaluate the regeneration potential of spent SBACs employed for adsorption of PC are very rare.
- Studies focusing on PC adsorptive performance on SBACs under continuous mode (that are more relevant for industrial applications) in both single and multi-pollutant aqueous systems to cover a wide range of PC of environmental concerns are lacking, thus they are recommended for future research.
- It is also recommended that the production processes and utilization of SBAC need to be economically re-evaluated and assessed within the realm of environmental sustainability via LCA analyses.

## Figures and Tables

**Table 1:** Physicochemical characteristics of raw sludge used in production of SBAC.

Sludge Type	Proximate Analysis							Ultimate Analysis										Ref.
	S_BET_ (m^2^ g^−1^)	Ash Content (wt % of Dried Matter)	Carbon (wt %)	Volatile Matter	Moisture Content	Particle Size	pH	C	H	N	S	O	Heavy Metals Cu Ni Pb Zn Hg					
PMS		20	34			600 um	-	34	5	-	0.24	41						[4]
ADWWTPS		22						57.7	8.5	9.3	0.5	24.0						[24]
VLS	2.9	22.0	39.4						5.6	6.4	0.9	19.8	306	76	64	634	<5	[25]
LS (40% lime)	4.8	57	27.9						3.5	2.9	0.9	18.7	201	32	49	320	<5	[25]
POES				34,000 mg/L		125 um	4.7						0.89			2.3		[26]
AGCWS	<3	23	48.7			0.1–0.25	6.9	48.7	7.5	9.4	0.6	10.8						[9]
Municipal DRAWS		20.4	41	65.9		10 mm												[27]
Municipal DUSS		32.6	6.8	60.6	82	3 mm												[28]
PMS		36.4	44		0.33	0.3 mm												[29]
WWTP	0.96	43.95	2.81	53.24	2.32		8.14											[30]
PMS		36.4				25 mm		44.8		0.4								[31]

**Table 2:** Typical total solid content after sludge dewatering treatment [14,21,32].

Sewage Treatment	Source of Sludge	Total Solids (%)
Centrifuge	Activated sludge	14–20
	Anaerobic digester	15–35
	Aerobic digester	8–10
Vacuum filter	Activated sludge	12–18
	Anaerobic digester (mixture)	17–23
Belt press	Activated sludge	12–18
	Anaerobic digester (mixture)	17–23
	Anaerobic digester	12–30
	Aerobic digester	12–25
Filter press	Activated sludge	27–33
	Anaerobic digester (mixture)	29–35

**Table 3:** Properties of Sludge-based Activated Carbon produced from thermal/physical treatment methods for PC adsorption.

Sludge Type	Carbonization Conditions				Physical Activation Conditions				Pre/Post Treatment	Textural Properties					Target Compound	Uptake (mg/g)	Ref.
	T (°C)	t (h)	HR (min^−1^)	atm	T (°C)	t (h)	HR (min^−1^)	atm		S_BET_ (m^2^/g)	V_micro_ (cm^3^/g)	V_meso_ (cm^3^/g)	Dp (nm)	pH			
VLS LS	600	1	Na Na na	N_2_ N_2_	Not activated Not activated					59	0.025			8.9	Phenol Phenol	170	[25]
	1000	1								96	0.036			10.6		182	
	600	1								33	0.01			12.4		161	
	800	1								62	0.015			12.5		185	
AGCWS	300	0.5	10	N_2_	Not Activated				Washed, dried/washed	10	-			7.6		-	[39]
	450									20							
	600									38							
	750									44							
AWWTPS	500 Microwave heating, 980 W	1	-	N_2_	Not Activated				Washed and dried/None	641	-	-	-	7	Hydroquinone	1218.3	[19]
		0.2	-	N_2_						540						1202.1	
AWWTPS AWWTPS + tyres (1:1)	650	0.5	40	na	Not activated				Washed and dried/None	60	0.04	0.05			Phenol	9.8	[51]
	650	0.5	40	na	Not activated				Washed and dried/None	59	0.03	0.08			Phenol	10.1	
POES	300	0.5		air	150	2	-	H_2_O	Washed and Dried/None						Phenol	-	[26]
	500	0.5		air			-									-	
	800	0.5		air			-									12.078	
	-	-		-			-									-	
FIS	500	1		Air	-	-	-	-	Dried/1 M HCl	380	-	-	-		4-bromophenol 2-bromophenol 2,4-dibromophenol	40.7 170.4 190.2	[52]
DMADS DRAWS DSBS	900 900 900 900 600	1	10 10 10 10	N_2_ N_2_ N_2_ N_2_ N_2_	- 838 - 838 875	- 1.21 - 1.34 1	- 0.7 g - 0.7 g 1.5 L	- Steam - Steam CO_2_	Sterilized-dried/None Sterilized-dried/None Sterilized-dried/None	125 155 180 265 90	0.05 0.06 0.07 0.11 0.03	0.11 0.08	4.4 4.5 2.7 3.5 2.5		Phenol	94 117.5 131 150 112	[42]
DMADS DRAWS	1000 - 950 250/500/1000 -	0.5	5 10 5 -	N_2_ N_2_ _-_	- 838 838 925 838 838 838 - 900 925	- 1.21 1.21 1 1.34 1.4 1.21 - 1.67 1	- 10 °C 10 °C 10 °C 10 °C - 10 °C 10 °C	- Steam Steam CO_2_ Steam Steam Steam - Steam CO_2_	Sterilized-dried/None Sterlized-dried/HCL (0 & 1 pH) Sterilized-dried/None Sterilized-dried/soaked in RO-24 h Sterilized-dried/HCL(3%) Sterilized-dried/None Sterilized-dried/None Sterilized-dried/None	153.4 179.3 na 169.1 268.9 497.4 269.11 8.1/12.1/150.1 214.4 227.8				8.9 7.6 8.1 6.1/7.3/9.8 10.1 8.4	Phenol	28.4% CWAO 62.7% 58.6% 65% 93% 62% 88/56/51 69.3 68.6	[53]
DRAWS	1000	na	10	N_2_	838	1.33	0.7 g with nitrogen	steam	Sterilized with steam/washed	265	0.11	0.17	3.5		Phenol	244.66	[27]
															P-Chloro phenol	216.2	
															p-nitro phenol	235	
DMADS	950	0.5	10	N_2_	838	1.21	10 °C	Steam	Sterilized-dried/None	269.1	-	-	-	8.1	Phenol	0.65~(5 g/L)	[54]
															o-Cresol	1	
															o-chlorophenol	0.82	
															p-nitrophenol	0.06	
AGCWS	None				700/800	0.5	10 °C	CO_2_	Washed/none	11/20	0.01/0.02		None	None	4-chloro phenol	-	[9]
						2				75/94	0.05/0.04					187/301.6	
						4				79/97	0.06/0.09					185.4/241.8	
					200/300/400	0.5	10 °C	Air		7/13/15	<0.01/0.01/0.03		None	None	4-chloro phenol	31.2/170.8/145.8	
						2				34/51/92	0.03/0.05/0.06					65.6/181.9/192.54	
						4				47/53/91	0.05/0.05/0.07					180.8/154.7/223.22	

**Table 4:** Properties of SBAC produced from chemical treatment method for PC adsorption.

Type of Sludge	Carbonisation Conditions				Chemical Activation Conditions					Pre/Post Treatment	Textural Properties					Target Compound	Uptake Capacity (mg/g)	Ref
	T (°C)	t (h)	HR (min^−1^)	atm	Reagents	T (°C)	t (h)	HR (min^−1^)	atm		S_BET_ (m^2^/g)	Vmicro (cm^3^/g)	Vmeso (cm^3^/g)	Dp (nm)	pH			
ADDDS & coconut husk (1:2)	500/600/700	2	15		ZnCl_2_ (3 M) ZnCl_2_ (5 M) ZnCl_2_ (7 M)	25 25 25	24 24 24			Dried/Dried-carbonized-HCL	448/648/425					phenol	5.2/5.2/4.0	[58]
	600	2	10								750						6.7	
	500/600/700	2	15								725/648/525						5.2/5.9/4.4	
	600	2	10								867						5.9	
	500/600/700	2	15								660/700/550						4.9/5.7/4.3	
	600	2	10								690						7.0	
PMS	800	2	20 °C	N_2_, 70 mL/min	ZnCl_2_:sludge = 3.5	85	8	Na	Na	Dried/22 h light exposure-carbonisation- and HCl-dried	1092		1.13	10	7	Phenol	-	[4]
AWWTPS	Not carbonised				H_2_SO_4_ (1:1) ZnCl_2_ (1:1)	650 650	0.5 0.083	40 5	NA	Dried/HCl	216 472			0.09 0.10		Phenol	24.8 88.16	[51]
PMS	700	1	15 °C	N_2_	ZnCl_2_ (2:1)	80	8	Na		Dried/HCl-dried	316.32	0.4357		6.124		phenol	15.58	[29]
PMS	560	0.41	20 °C	N_2_	ZnCl_2_ (0.9:1)	80	6			Dried/HCl-dried	907.20	0.42		3.13	4.6	Phenol 4-Nonyl phenol 2-chloro phenol	370 296 325	[31]
DS	600	1	-	-	ZnCl_2_ (40%)	RT	24		Na	HCl	195	0.06	0.14	3.5		phenol	45.12	[28]
DS	500	1	20 °C	N_2_	0–2 M citric acid and 0.5 M ZnCl_2_	RT	24			Dried/carbonisation-HCL-dried	792.4					4-chloro phenol Phenol	372.94 189.16	[61]
AWWTPS	625	0.5	40 °C	N_2_	H_2_SO_4_ (1:1)	Na	48	NA	NA	Dried/carbonisation-HCL-dried	390	NA		0.12/0.5 0.12		Phenol (Indigo carmine + phenol)	42.04/29.46 10.2	[59]
DWWTPS	650	1	10 °C	NA	3 M H_2_SO_4_ (1:1)	Na	48			Dried/carbonised-HCl-Dried	166.20	Na		Na	5.5	2-chlor phenol	47.98	[30]
WBS					H_2_SO_4_	700	0.5	NA	NA	Dried/carbonised	253	0.08		Na	Na	Phenol	10	[24]
AGCWS	Not carbonised				KOH (1:1)	450/750	0.5	10	N_2_	Dried/HCl	131/950	<0.01/0.40	0.12/0.23			4-chloro phenol	140.8/170.6	[9]
					KOH (3:1)	450/750	0.5	10	N_2_		262/1832	0.01/0.75	0.16/0.36				146.54/265.08	
DMADS	Not carbonised				K_2_CO_3_ (1:1)	800	1	18–20	N_2_	Dried/washed with water Dried/washed with HCL (5%)	421.8 863.8				8.2 5.2	Phenol	Oxidation 87.1% (5 g/L) 93.2%	[53]
DMADS	Not carbonised				K_2_CO_3_ (1:1)	800	1	18-20	N_2_	Dried/washed with water	421.8				8.2	Phenol o-Cresol o-chlorophenol p-nitrophenol	Oxidation 87.1% (5 g/L) 0.88 0.83 0.06	[54]
WWTPS	1000	1	5 °C	N_2_, 50 mL/min	NaOH (1:1)	500/600/800	2	5	N_2_	Carbonised-HCl/washed-dried	319/346/307	0.438/0.465/0.403		17.2/12.3/14	NA	phenol	-/96.15/-	[44]

**Table 5:** Comparison of properties and adsorption of dried activated sludge used for phenolic compounds adsorption.

Type of Sludge	Drying Conditions	Diameter (mm)	Adsorbate	Temperature (°C)	pH	Model Used	Uptake Capacity (mg/g)	Ref
PMS	60/24 h	0.006 mm	*o*-Chlorophenol	25	1	Langmuir	281.1	[92]
			*p*-Chlorophenol	25	1		287.2	
DAS		-	Phenol	25	1.0	Langmuir/Freundlich	91.0	[94]
AAS	60/24 h	-	Phenol		1.0	Langmuir/Freundlich	180.9	[95]
WWTPS	HNO_3_ washed and rinse with 0.1 NaCl		Phenol	NA	7	None	0.06	[96]
AWWTP	60/24 h	NA	Phenol	40	8	Freundlich	42.7	[97]
AGS	Dried	NA	4-Chlorophenol	25	3.6	Langmuir/Freundlich	7.77	[90]
AAS	60/24 h	0.775	Phenol	30	NA	Langmuir	90.5421	[98]
			Binary(phenol + Pb)	30		Langmuir	30.7843	
SSTP 5% (*w*/*v*)	60/24 h	<0.1 mm	Phenol	NA	6–8	None	17.3 from 100 ppm phenol	[99]
AS (50%) MR	NA	1	Phenol	NA	1.0	Breakthrough curves	9.0	[100]
ASB	105/6 h	NA	Nonylphenol	22	NA	Freundlich	90% removal from 4.15 mg/L	[101]

**Table 6:** Comparison of Langmuir and Freundlich isotherm parameters for adsorption of phenolic compounds by various SBACs produced by different methods.

Type of Sludge	Activation Method	S_BET_	Phenolic Compound	Langmuir			Freundlich			Ref
				Qm (mg/g)	A (L/mg)	*R*^2^	K_F_	*n*	*R*^2^	
DASS	KOH (3:1)	1832	4-Chlorophenol	265.8	0.0156	0.994				[9]
	CO_2_ (800)	94	4-Chlorophenol	301	0.0014	0.972				
	Air (400)	91	4-Chlorophenol	22.96	0.00169	0.965				
AWWTPS	H_2_SO_4_ (1:1)	390	Phenol	42.04	0.02	0.969	6.33	3.51	0.9748	[59]
WWTPS	NaOH (1:1	346	Phenol	96.154	0.128	0.979	18.065	2.48	0.989	[44]
AWWTPS	ZnCL_2_ (40%)	195.28	Phenol	18.3	0.114	-				[120]
			2-Chlorophenol	51.8	0.118	-				
			4-Chlorophenol	58.1	0.129	-				
			2,4-Dichlorophenol	137.0	0.162	-				
DWWTPS	3 M H_2_SO_4_ (1:1)	166.20	2-Chlorophenol	47.977	0.485	0.918	18	4.18	0.977	[30]
DWWTPS	3 M H_2_SO_4_ (1:1)	162.2	Phenol	26.16	0.109	0.927	6.059	3.02	0.996	[60]
DRAWS	838-steam	265	Phenol	244.4	0.0007	0.972	0.009	0.469	0.992	[27]
			*p*-Chlorophenol	216.2	0.00967	0.990	0.004	0.144	0.879	
			*p*-Nitrophenol	235.5	0.00095	0.559	0.841	1.354	0.925	
			*p*-Hydroxybenzoic acid	150.4	0.00095	0.175	0.031	0.741	0.889	
PMS	ZnCL_2_ (0.9:1)	907.20	Phenol	370.4	0.008	0.988	9.897	1.688	0.957	[31]
			4-Nitrophenol	296.1	0.0631	0.993	53.75	2.935	0.960	
			2-Chlorophenol	325.1	0.0249	0.994	26.58	2.144	0.944	
CFS	N_2_ (750)	44	4-Chlorophenol	37.88	0.004	0.992	0.0012	2.027	0.968	[39]
PMS	ZnCL_2_ (1:3.5)	1092	Phenol	None	None	None	0.44	1.149	NA	[4]
DUSS	ZnCL_2_ (40%)	195	Phenol(W)	45.12	38.8	0.665	0.044	1.26	0.978	[28]
			Phenol(C)	49.25	0.402	0.675	0.143	1.40	0.667	
PMS	ZnCL_2_ (2:1)	316.32	Phenol	15.585	1.0185	0.962	7.3781	3.534	0.996	[29]
				44.4 (LF)	0.013 (LF)	0.998 (LF)				
VLS	600 N_2_	59	Phenol	170	0.0022	0.975	4.9	1.29	0.961	[25]
	1000 N_2_	96		182	0.0051	0.988	0.2	0.617	0.936	
LS	600 N_2_	33	Phenol	161	0.0032	0.862	0.6	0.75	0.888	[25]
	800 N_2_	62		185	0.0034	0.897	0.5	0.74	0.826	
POES	800(Air)	NA	Phenol	12.078	0.069	0.957	2.048	2.79	0.999	[26]

## Abbreviations

AAS	Aerobic activated sludge
AGS	anaerobic granular sludge
AAS	Anaerobic activated sludge
ADWWTPS	Aerobically digested WWTP sludge
ADDWWTP	Anaerobically digested and dewatered sludge
AGCWS	Aerobic granular sludge from cosmetic factory
ASB	Activated sludge biomass
AS (50%) MR	Activated sludge (50%) immobilized in Mowital^®^ B30H resin
ASS	Activated Sludge System
AWWTPS	Anaerobic wastewater treatment plant sludge
CFS	Cosmetic factory sludge
DAEDS	Dewatered aerobically digested sludge
DASS	Dried aerobic sewage sludge
DMADS	Dewatered anaerobically digested sludge
DSBS	Dewatered secondary biological sludge
MDSS	Mechanical dewatered sewage sludge
DUSS	Dewatered undigested sewage sludge
DRAWS	Dewatered raw sludge
FIS	Fertilizer industry sludge
GAC	Granular activated carbon(s)
LS	Limed sludge
LCA	Life cycle assessment
PC	Phenolic compounds
POES	Palm oil effluent sludge
S_BET_	Adsorbent specific surface area measured using Brunauer Emmett Teller (BET) method
SBAC	Sludge based activated carbon
SSTP	sewage sludge from sludge treatment plant
VLS	Viscous liquid sludge
WBS	Waste biological sludge
WWTP	Wastewater treatment plant
WWTPS	Wastewater treatment plant sludge
